# Genome-Wide Identification and Analysis of *SUS* and *AGPase* Family Members in Sweet Potato: Response to Excessive Nitrogen Stress during Storage Root Formation

**DOI:** 10.3390/ijms25158236

**Published:** 2024-07-28

**Authors:** Shaoxuan Han, Yanhui Lin, Yayi Meng, Chengcheng Si

**Affiliations:** 1 School of Tropical Agriculture and Forestry (School of Agricultural and Rural, School of Rural Revitalization), Hainan University, Danzhou 571700, China; 20213007002@hainanu.edu.cn (S.H.); 21220951310162@hainanu.edu.cn (Y.M.); 2 School of Breeding and Multiplication (Sanya Institute of Breeding and Multiplication), Hainan University, Sanya 572025, China; 3 Hainan Key Laboratory of Crop Genetics and Breeding, Institute of Food Crops, Hainan Academy of Agricultural Sciences, Haikou 571100, China; lyh_1012@163.com

**Keywords:** excessive nitrogen stress, sweet potato, storage root, starch synthesis, *IbAGPL3*

## Abstract

(1) The development of sweet potato storage roots is impacted by nitrogen (N) levels, with excessive nitrogen often impeding development. Starch synthesis enzymes such as sucrose synthase (SUS) and ADP-glucose pyrophosphorylase (AGPase) are pivotal in this context. Although the effects of excessive nitrogen on the formation of sweet potato storage roots are well documented, the specific responses of *IbSUSs* and *IbAGPases* have not been extensively reported on. (2) Pot experiments were conducted using the sweet potato cultivar “Pushu 32” at moderate (MN, 120 kg N ha^−1^) and excessive nitrogen levels (EN, 240 kg N ha^−1^). (3) Nine *IbSUS* and nine *IbAGPase* genes were categorized into three and two distinct subgroups based on phylogenetic analysis. Excessive nitrogen significantly (*p* < 0.05) suppressed the expression of *IbAGPL1*, *IbAGPL2*, *IbAGPL4*, *IbAGPL5*, *IbAGPL6*, *IbAGPS1*, and *IbAGPS2* in fibrous roots and *IbSUS2*, *IbSUS6*, *IbSUS7*, *IbSUS8*, *IbSUS9*, *IbAGPL2*, and *IbAGPL4* in storage roots, and then significantly (*p* < 0.05) decreased the SUS and AGPase activities and starch content of fibrous root and storage root, ultimately reducing the storage root formation of sweet potato. Excessive nitrogen extremely significantly (*p* < 0.01) enhanced the expression of *IbAGPL3*, which was strongly negatively correlated with the number and weight of storage roots per plant. (4) *IbAGPL3* may be a key gene in the response to excessive nitrogen stress and modifying starch synthesis in sweet potato.

## 1. Introduction

Sweet potato (*Ipomoea batatas* (L.) Lam., 2n = 6x = 90) is an autohexaploid crop within the family Convolvulaceae, specifically classified under the genus *Ipomoea* and section *Batatas* [1,2]. It holds the position of the third most important tuberous crop after cassava and potato [3,4]. As a carbohydrate-rich crop, sweet potato is a crucial source of starch and its derivatives, widely used in food, biofuel, and various industrial applications [5]. The synthesis and accumulation of starch significantly influence the development of sweet potato storage roots [6]. Over the past five decades, there has been more than a 100-fold increase in global urea application [7]. Nevertheless, plants absorbed only about half of the applied urea, leading to excess nitrogen in the soil and exceeding the absorption capacity of the plants [8]. As a result, excessive nitrogen became an abiotic stressor, resulting in decreases in the yield and quality of crops [9]. Research indicates that optimal nitrogen application can enhance the starch content of storage roots [10] and yield [11,12,13] of sweet potato. Conversely, excessive nitrogen application can reduce the starch content, resulting in fewer and earlier storage roots, ultimately decreasing overall yield [12,13,14,15,16]. Sucrose synthase (SUS; EC 2.4.1.13) serves as a critical enzyme in the starch synthesis pathway of plants, converting sucrose into UDP-glucose and fructose, which are essential substrates for starch production [1]. The enzyme ADP-glucose pyrophosphorylase (AGPase; EC 2.7.7.27) acts as the primary regulatory and rate-limiting enzyme in starch synthesis [2,17,18], generating ADP-glucose, a crucial substrate for this process [19].

SUS proteins (SUSs) are encoded by a small multigene family, referred to as the *SUS* family, and are vital in a variety of plants [1]. Most plant species possess at least three *SUS* genes [20], with numerous *SUS* genes identified across diverse plant genomes, including *Arabidopsis thaliana*, rice, and *Lotus japonicus* [21,22,23]. In *Arabidopsis thaliana*, six *SUS* genes were identified [21], with sucrose synthases 2 and 3 involved in metabolic homeostasis and directing carbon toward starch synthesis in developing seeds [24]. In rice, *SUS3* and *SUS4* are predominantly expressed in the caryopsis [22], whereas in cotton, *GaSUS7* is found in the stems and petals [20], and in *LjSUS4*, it is exclusively expressed in the flowers [23]. The *SUS3* gene in potato is highly expressed in stems and roots, while *SUS4* is primarily active in the storage and vascular tissues of tubers [1]. The overexpression of *MeSUS4.2* in the fibrous roots of cassava leads to suppressed storage root formation, and the inhibition of *MeSUS4.1* expression results in reduced storage root yield [17]. Optimal nitrogen application could enhance starch accumulation by upregulating *SUS* and *malZ* expression in grains, increasing the starch content in both waxy and non-waxy proso millets [25]. However, excessive nitrogen significantly reduced SUS enzyme activity, decreasing starch biosynthesis and grain weight in rice [9], and downregulated the sugar-metabolism-related gene (*MdSUS5*) in apple, lowering the soluble sugar content in leaves [26]. In sweet potato, nine *SUS* genes were identified, with *IbSUS2*, *IbSUS5* and *IbSUS7* likely playing crucial roles in starch biosynthesis and storage root development [1]. The response of *IbSUS* genes to excessive nitrogen stress during sweet potato storage root formation, however, has not been previously documented.

*AGPases* are genes that regulate AGPase activity and can be classified as *APLs* or *APSs* in plants [27]. Genome-wide analyses have revealed various numbers of *APLs* and *APSs* in *Arabidopsis thaliana* [28], rice [29,30], and cassava [31]. For example, in *Arabidopsis thaliana*, four genes encoded large subunits (LSs) and two genes encoded small subunits (SSs). It has been established that *APS1* is crucial for starch biosynthesis [28]. In rice, *OsAGPS2a* and *OsAGPS2b* are located in the chloroplasts of leaves and the cytosol of the endosperm, respectively [30]. In cassava, the *MeAGPL1.3* gene shows the highest expression in storage roots, while *MeAGPL1.3* is expressed in storage roots only [31]. Five out of the six *AGPases* in the grains of waxy and non-waxy proso millets were significantly downregulated following nitrogen application, potentially enhancing carbon flux into starch [25]. Li et al. reported that AGPase activity decreased with excessive nitrogen levels in potato [32]. Additionally, excessive nitrogen application significantly reduced AGPase activity, thereby decreasing starch biosynthesis and grain weight in inferior spikelets in rice [9]. In sweet potato, the *AGPase* gene family is vital for starch synthesis [2]. Both *IbAGPS1* and *IbAGPS2* showed their highest expression in storage roots, with *IbAGPS1* being generally expressed at higher levels than *IbAGPS2* in all tissues, especially in harvestable storage roots [33]. SSs exhibited the highest expression levels in storage roots [33]. Duan et al. found that excessive nitrogen inhibited starch synthesis in roots by downregulating *AGPa* and *AGPb* expression in sweet potato [12]. However, the identification of *AGPase* genes across the entire genome and the regulatory mechanisms of *AGPase* on starch synthesis under excessive nitrogen stress during sweet potato storage root formation remain unclear.

In this research, the *SUS* and *AGPase* gene families in sweet potato were identified and analyzed based on the entire genome. This study also determined the starch content, SUS and AGPase activities, and the expression levels of *IbSUSs* and *IbAGPases* in sweet potato fibrous roots and storage roots under excessive nitrogen stress. This study offers new insights into the mechanisms regulating the expression of *IbSUSs* and *IbAGPases* due to excessive nitrogen stress during the formation of storage roots in sweet potato.

## 2. Results

### 2.1. Identification and Analysis of IbSUSs and IbAGPases

**Figure 1:**
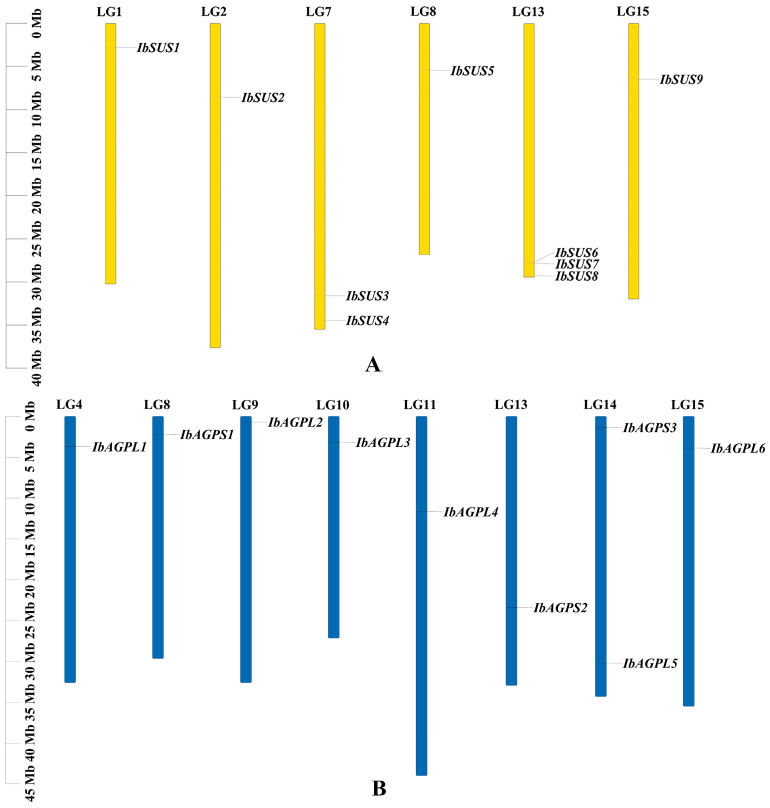
Chromosomal localization and distribution of *SUSs* (**A**) and *AGPases* (**B**) in sweet potato. The bars represent chromosomes. The chromosome numbers are shown above, and the gene names are displayed on the right side. The relative chromosomal localization of each *SUS* and *AGPase* gene is marked on the gray line of the right side and indicated by the unit Mbp.

To achieve a comprehensive identification of *SUSs* and *AGPases* in sweet potato, three primary methods were employed: HMM search, blast search, and the CD-search database. The results indicate that the sweet potato *SUS* and *AGPase* families each contain nine members, designated as “*IbSUSs*” and “*IbAGPases*”. The core properties of these proteins were examined using sweet potato sequences (Table 1). The predicted length of IbSUS proteins ranged from 743 to 906 aa, with molecular weight (MW) spanning from 84,781.48 to 101,580.88 Da. The isoelectric point (pI) ranged from 6.02 to 6.99, suggesting that IbSUSs were all acidic amino acids. Except for IbSUS1, which had an instability index of 40.26, all of the other IbSUSs were stable, with instability index between 33.53 and 39.82. The grand average of hydropathicity (GRAVY) values of IbSUSs varied from −0.351 to −0.202, indicating their hydrophilic nature. Predictions for subcellular localization revealed that IbSUS1, IbSUS3, IbSUS4, and IbSUS9 were located in the cytosol, IbSUS2 and IbSUS8 in chloroplasts, IbSUS6 and IbSUS7 in mitochondria, and IbSUS5 on the plasma membrane (Table 1). Chromosomal localization showed that *IbSUSs* were unevenly distributed across six chromosomes. Specifically, three *IbSUSs* were located on LG13 (*IbSUS6*, *IbSUS7*, *IbSUS8*), two on LG7 (*IbSUS3*, *IbSUS4*), and one each on LG1 (*IbSUS1*), LG2 (*IbSUS2*), LG8 (*IbSUS5*), and LG15 (*IbSUS9*) (Figure 1A). Additionally, *IbSUS6* and *IbSUS7* were found in gene clusters (Figure 1A), suggesting that tandem duplication had played a significant role in the expansion of the *IbSUS* gene family. The *AGPase* gene family includes six large subunit genes (*IbAGPL*) and three small subunit genes (*IbAGPS*). The encoded amino acid length, MW, and pI of IbAGPases ranged from 484 to 827 aa, 53,623.32 to 91,001.22 Da, and 5.78 to 9.08, respectively. Eight IbAGPases were stable, with instability index below 40, while IbAGPS3 was unstable, showing an instability index of 49.69. The GRAVY values of IbAGPases ranged from −0.287 to −0.154, indicating their hydrophilic nature. Subcellular localization analysis predicted that IbAGPL2, IbAGPL5, and IbAGPS1 were in chloroplasts; IbAGPL1 was in the endoplasmic reticulum; IbAGPL6 was associated with the cytoskeleton; and IbAGPL2 was present in both chloroplasts and cytosol (Table 1). Chromosomal localization results revealed that *IbAGPase* genes were unevenly distributed across eight different chromosomes; two genes were located on LG14 (*IbAGPL5*, *IbAGPS3*), and one on LG4 (*IbAGPL1*), LG8 (*IbAGPS1*), LG9 (*IbAGPL2*), LG10 (*IbAGPL3*), LG11 (*IbAGPL4*), LG13 (*IbAGPS2*), and LG15 (*IbAGPL6*) (Figure 1B).

### 2.2. Analysis on Conserved Motif and Gene Structure of IbSUSs and IbAGPases

**Figure 2:**
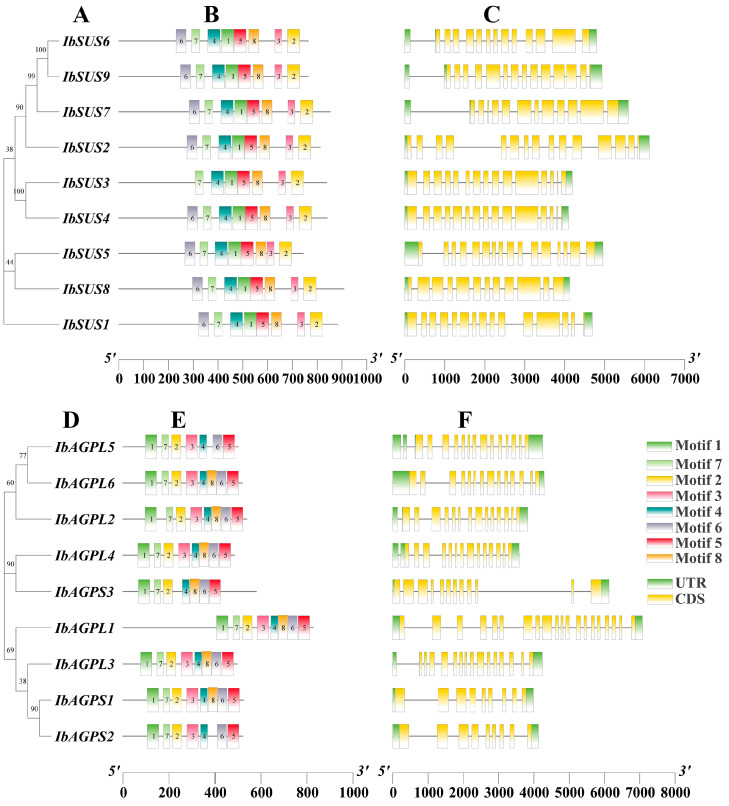
Phylogenetic relationship, conserved motifs, and gene structure analysis of *IbSUSs* and *IbAGPases*. (**A**,**D**) The phylogenetic trees of *IbSUSs* (**A**) and *IbAGPases* (**D**) were constructed using MEGA 11; (**B**,**E**) motif distribution of the IbSUS (**B**) and IbAGPase (**E**) proteins. The conserved motifs of IbSUS and IbAGPase proteins were determined by MEME (http://meme-suite.org/tools/meme (accessed on 1 July 2023)) and visualized by TBtools v2.096. The motifs, numbered 1–8, are displayed in different colored boxes; (**C**,**F**) gene structures of *IbSUSs* (**C**) and *IbAGPases* (**F**). The yellow boxes and gray lines represent exons and introns, respectively.

To comprehend the conserved motifs of IbSUSs (Figure 2B) and IbAGPases (Figure 2E), the MEME suite was employed to examine sequence motifs. We identified eight conserved motifs. For IbSUS3 (Figure 2B), IbAGPL5, IbAGPS2, and IbAGPS3 (Figure 2E), there were seven conserved motifs, while the remaining eight IbSUSs exhibited eight conserved motifs with similar distribution patterns (Figure 2B) and the additional six IbAGPases also presented eight conserved motifs with analogous distribution (Figure 2E). Typically, exon/intron configurations are conserved among homologous genes within a gene family [1]. To gain a deeper insight into the evolution of the *IbSUS* and *IbAGPase* gene families, we analyzed the exon–intron arrangements of these genes. The number of exons in *IbSUSs* varied slightly, ranging from 12 (*IbSUS7*, *IbSUS8*) to 20 (*IbSUS5*) (Figure 2C). For *IbAGPases*, exon numbers varied from 9 (*IbAGPS1*, *IbAGPS2*) to 20 (*IbAGPL1*) (Figure 2F).

### 2.3. Analysis of Cis-Acting Element in the Promoters of IbSUSs and IbAGPases

**Figure 3:**
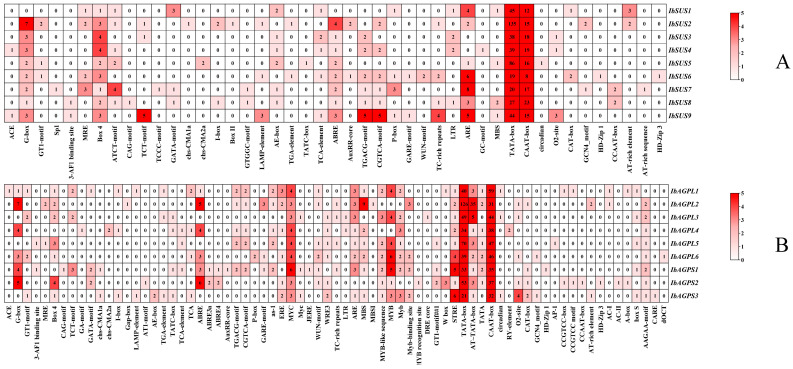
Cis-acting element analysis in the promoters of *IbSUSs* (**A**) and *IbAGPases* (**B**).

We explored the regulatory elements in the upstream regions of *IbSUSs* (Figure 3A) and *IbAGPases* (Figure 3B), analyzing 2000 bp upstream sequences to elucidate their regulatory roles. Our study identified that all of the *IbSUSs* promoters included substantial core/binding elements including TATA-box and CAAT-box. Each *IbSUS* promoter contained at least one development element. For instance, we found the O2-site (zein metabolism regulatory element) in the promoters of *IbSUS3*, *IbSUS4*, and *IbSUS9*. Box 4, G-box, and AE-box, all of which belonged to the light-responsive elements, existed in most of the *IbSUS* promoters. The *IbSUS* promoters contained plentiful hormone-responsive elements, such as ABA-responsive element ABRE, JA-responsive elements CGTCA-motif and TGACG-motif, and SA-responsive elements TATC-box. Furthermore, most of the *IbSUS* promoters contained abiotic/biotic elements such as antioxidant response element ARE (Figure 3A). We could suggest that *IbSUSs* are involved in the regulation of plant growth and development, hormone crosstalk, and abiotic/biotic stress adaptation, and *IbSUSs* may play more important roles in development and the light response.

Our analysis revealed numerous core/binding elements, including TATA-box and CAAT-box, within the *IbAGPase* promoters. The *IbAGPL2* promoters also had multiple AT-TATA-boxes. Every *IbAGPase* promoter contained at least one development element. For instance, the O2-site, associated with zein metabolism, was found in the promoters of *IbAGPL2*, *IbAGPL3*, *IbAGPL6*, *IbAGPS2*, and *IbAGPS3*. The CAT-box, linked to meristem formation and cell division, was present in the promoters of *IbAGPL2*, *IbAGPS1*, *IbAGPS2*, and *IbAGPS3*. The GCN4_motif, which controls seed-specific expression, was identified in the promoters of *IbAGPL6* and *IbAGPS3*. Additionally, most *IbAGPase* promoters contained light-responsive elements such as Box 4 and G-box. These promoters also harbored ample hormone-responsive elements, including ABRE, ABRE4, and ABRE3a (ABA-responsive), CGTCA-motif and TGACG-motif (JA-responsive), as well as TCA and TATC-box (SA-responsive). Drought-responsive elements like MYB and MYC, salt-responsive element MBS, and antioxidant response element ARE were also abundant (Figure 3B). The results indicate that *IbAGPases* play a critical role in regulating plant growth and development, hormone signaling, and responses to abiotic and biotic stress, possibly having a substantial impact on plant development and light response.

### 2.4. Analysis of Phylogenetic Relationship of SUSs and AGPases

**Figure 4:**
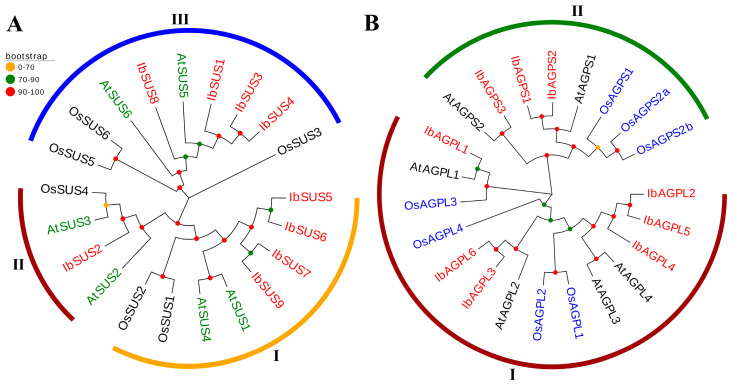
Phylogenetic analysis of *SUSs* (**A**) and *AGPases* (**B**) in sweet potato, *Arabidopsis thaliana*, and *Oryza sativa*. (**A**) Based on the evolutionary distance, a total of 21 *SUSs* were divided into three groups (Groups I, II, and III, with orange, dark red, and blue, respectively). The red color represents nine *IbSUSs* in sweet potato. The green represents six *AtSUSs* in *Arabidopsis thaliana*. The black represents six *OsSUSs* in *Oryza sativa*. (**B**) A total of 22 *AGPases* were divided into two groups (Groups I and II, with dark red and green, respectively). The red color represents nine *IbAGPases* in sweet potato. The black represents six *AtAGPases* in *Arabidopsis thaliana*. The blue represents seven *OsAGPases* in *Oryza sativa*.

To explore the evolutionary relationships of *SUS* and *AGPase* genes, we constructed phylogenetic trees including twenty-one *SUS* genes (nine from sweet potato, six from *Arabidopsis thaliana*, and six from *Oryza sativa*) (Figure 4A) and twenty-two *AGPase* genes (nine from sweet potato, six from *Arabidopsis thaliana*, and seven from *Oryza sativa*) (Figure 4B). We grouped all *SUS* genes into three distinct groups based on their evolutionary distances. The distribution pattern of *SUS* genes was as follows. Group I included eight genes (four from sweet potato, two from *Arabidopsis thaliana*, and two from *Oryza sativa*); Group II included four genes (one from sweet potato, two from *Arabidopsis thaliana*, and one from *Oryza sativa*); Group III included nine genes (four from sweet potato, two from *Arabidopsis thaliana*, and three from *Oryza sativa*) (Figure 4A). Similarly, *AGPase* genes were divided into two groups. The distribution of *AGPase* genes was as follows. Group I included fourteen genes (six from sweet potato, four from *Arabidopsis thaliana*, and four from *Oryza sativa*); Group II included eight genes (three from sweet potato, two from *Arabidopsis thaliana*, and three from *Oryza sativa*) (Figure 4B).

### 2.5. Synteny Analysis of SUSs and AGPases

**Figure 5:**
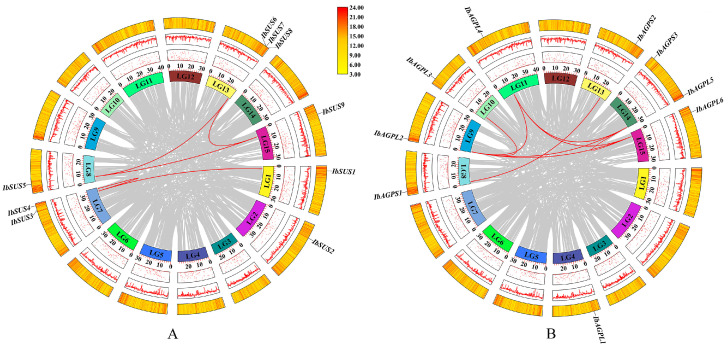
Schematic representations of the chromosomal distribution and interchromosomal relationships among sweet potato *SUS* (**A**) and *AGPase* (**B**) genes. Chromosomes are represented in different colors. Red lines between *IbSUS* (**A**) or *IbAGPase* (**B**) genes represent segmental duplication events that occurred in the sweet potato *SUS* (**A**) and *AGPase* (**B**) gene families.

**Figure 6:**
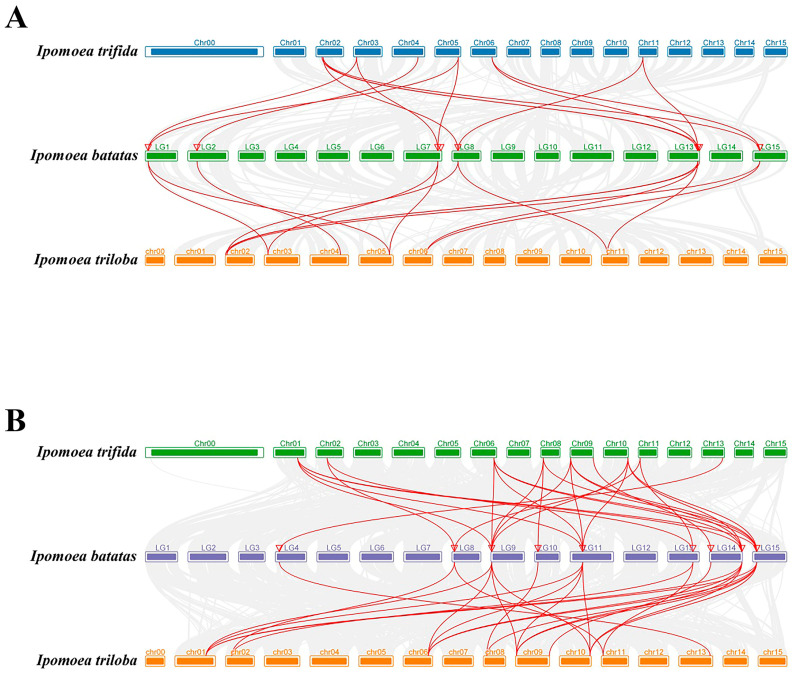
Synteny analyses of *SUS* (**A**) and *AGPase* (**B**) genes between sweet potato and other representative plant species (*Ipomoea trifida* and *Ipomoea triloba*). Gray lines indicate significantly collinear blocks within and among plant genomes. Red lines indicate significantly collinear blocks within and among plant genomes between *SUS* (**A**) and *AGPase* (**B**) genes. The red triangles represent the position of *IbSUS* (**A**) and *IbAGPase* (**B**) genes on the chromosome of sweet potato.

To identify duplication events, we examined tandem *SUS* and *AGPase* duplicated genes. Our analysis revealed five pairs of tandem *SUS* duplicated genes (Figure 5A) and eight pairs of tandem *AGPase* duplicated genes (Figure 5B) on sweet potato chromosomes. This suggested that some *IbSUS* and *IbAGPase* genes might have originated from gene duplication, with tandem duplication significantly aiding the expansion of the *IbSUS* and *IbAGPase* families. Moreover, the Ka/Ks values of the syntenic gene pairs in our study were all below 1 (Table 2), indicating that the *IbSUS* and *IbAGPase* gene families underwent strong purifying selection during evolution. Synteny analysis is a critical strategy in comparative genomics, playing a key role in assessing molecular evolutionary relationships among species [34]. We constructed comparative syntenic maps to demonstrate the connections between *SUS* and *AGPase* genes in sweet potato and two wild sweet potato species (*Ipomoea trifida* and *Ipomoea triloba*), thereby enhancing our understanding of the phylogenetic relationships within the *SUS* and *AGPase* families (Figure 6). For *SUS* (Figure 6A) and *AGPase* (Figure 6B) gene families analyzed in this study, corresponding sequences from each gene in two wild species and cultivated sweet potato were also found in other sweet potato varieties, indicating that these genes were highly conserved in sweet potato.

### 2.6. Protein Interaction Network of IbSUSs and IbAGPases

**Figure 7:**
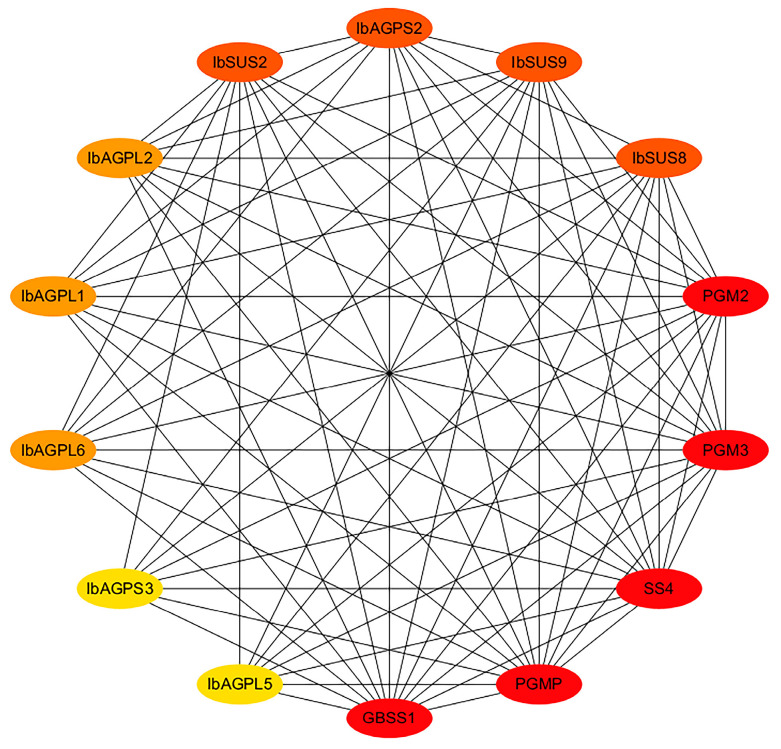
Functional interaction networks of IbSUSs and IbAGPases in sweet potato according to orthologues in *Arabidopsis*. Network nodes represent proteins, and lines represent protein–protein associations. The depth of the color represents its importance in the network, and the darker the color, the more important it is.

To investigate the regulatory relationships of IbSUSs and IbAGPases, we established an interaction network based on Arabidopsis orthologous proteins. The findings revealed that IbSUS2, IbSUS8, and IbSUS9 could interact with starch granule-bound synthase (GBSS1) and phosphoglucomutase enzymes (PGMP, PGM2, and PGM3). Similarly, IbAGPL1, IbAGPL2, IbAGPL5, IbAGPL6, IbGPS2, and IbGPS3 were found to interact with GBSSI and the same phosphoglucomutase enzymes (PGMP, PGM2, and PGM3) (Figure 7). These results highlight the significant involvement of IbSUSs and IbAGPases in sweet potato starch metabolism.

### 2.7. Prediction of Secondary and Tertiary Structure of SUSs and AGPases

**Figure 8:**
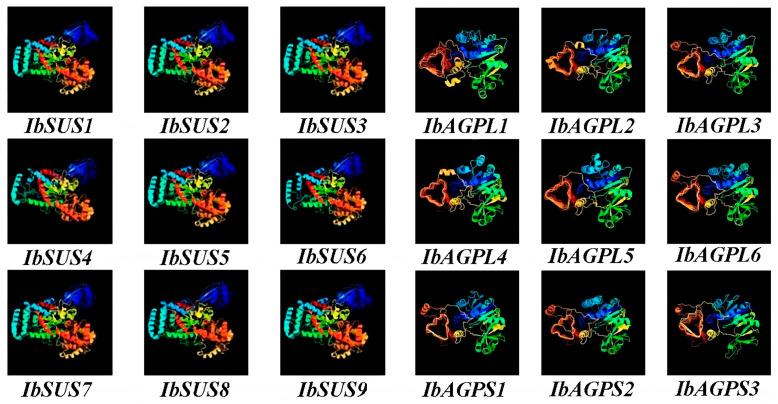
Homologous modeling prediction of the tertiary structure of the conserved domain of IbSUSs and IbAGPases.

Our analysis of secondary structures revealed that IbSUSs predominantly exhibit α-helix (48.99% to 53.35%) and random coil (26.61% to 32.50%), while IbAGPases mostly consist of random coil (49.09% to 61.20%) and α-helix (19.96% to 35.91%) (Table 3). Predictions of tertiary structure indicated that the proteins of the SUS and AGPase families in sweet potato show a high degree of structural similarity (Figure 8). The specific structural features identified are closely related to their functional roles, suggesting that the unique structural domains are essential for the functions of the *IbSUS* and *IbAGPase* gene families.

### 2.8. Starch Synthesis

**Figure 9:**
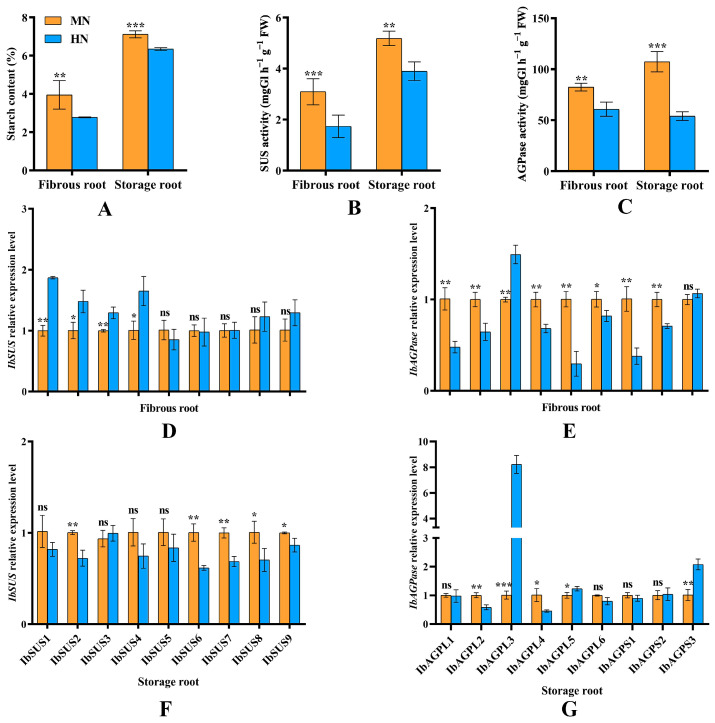
(**A**) Starch content in fibrous root and storage root in sweet potato; (**B**,**C**) SUS and AGPase activity in fibrous root and storage root in sweet potato; (**D**–**G**) relative expression levels of *IbSUS* and *IbAGPase* genes in response to excessive nitrogen stress. MN, 120 kg ha^−1^ N; EN, 180 kg ha^−1^ N. *: *p* < 0.05; **: *p* < 0.01; ***: *p* < 0.001; ns: not significant. Error bars indicate the standard derivations.

Compared to MN treatment, EN treatment significantly (*p* < 0.05) reduced the starch content in both fibrous and storage roots (Figure 9A). This reduction was primarily due to a significant (*p* < 0.05) decrease in the activity levels of SUS (Figure 9B) and AGPase (Figure 9C) in these roots. Furthermore, expression of most *IbSUSs* (*IbSUS1*, *IbSUS2*, *IbSUS3*, and *IbSUS4*) was significantly enhanced by EN in fibrous roots, whereas it was decreased in storage roots. Moreover, EN treatment significantly (*p* < 0.05) inhibited the expression of *IbAGPL1*, *IbAGPL2*, *IbAGPL4*, *IbAGPL5*, *IbAGPL6*, *IbAGPS1*, and *IbAGPS2* in fibrous roots during their transition from adventitious to storage roots (Figure 9E). On the other hand, the expression of *IbSUS2*, *IbSUS6*, *IbSUS7*, *IbSUS8*, *IbSUS9* (Figure 9F), *IbAGPL2*, and *IbAGPL4* (Figure 9G) in the storage roots of EN-treated plants decreased significantly (*p* < 0.05) when the number of storage roots stabilized at 45 days post-planting. Notably, *IbAGPL3* expression was highest in the fibrous (Figure 9E) and storage roots (Figure 9G) under EN treatment, showing highly significant (*p* < 0.01) differences.

### 2.9. Agronomic Traits under Enclosure Condition

**Figure 10:**
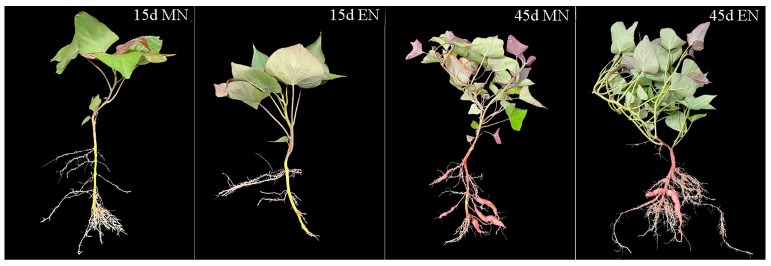
Sweet potato growth during storage root formation. MN, 120 kg ha^−1^ N; EN, 180 kg ha^−1^ N.

Excessive nitrogen application negatively impacted root development and storage root formation in sweet potato (Figure 10). The adventitious root number under EN treatment was considerably lower than MN treatment during adventitious root differentiation into storage roots of sweet potato (15 d). Additionally, the storage root number and storage root weight per plant under EN treatment remained significantly (*p* < 0.05) lower than MN treatment at 45 d when the storage root number stabilized (Table 4).

### 2.10. Relationship between IbAGPL3 Gene Expression Level and Agronomic Traits

**Figure 11:**
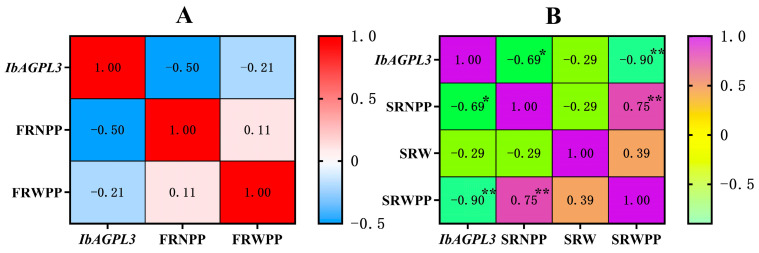
Correlation analysis between *IbAGPL3* gene expression level and agronomic traits. (**A**) Correlation analysis between *IbAGPL3* gene expression level in fibrous root and fibrous root number per plant (FRNPP), fibrous root weight per plant (FRWPP). Red represents positive correlation, and light blue represents negative correlation. (**B**) Correlation analysis between *IbAGPL3* gene expression level in storage root and storage root number per plant (SRNPP), storage root weight (SRW), storage root weight per plant (SRWPP). Purple represents positive correlation, and light green represents negative correlation. *: *p* < 0.05; **: *p* < 0.01.

A correlation analysis was carried out to analyze the relation between *IbAGPL3* expression, fibrous root number per plant (FRNPP), fibrous root weight per plant (FRWPP), storage root number per plant (SRNPP), storage root weight (SRW), storage root weight per plant (SRWPP), and the results displayed the following patterns (Figure 11). First, the fibrous root number and weight per plant negatively correlated with the expression of *IbAGPL3* in fibrous root (r > 0). Second, there were negative correlations among storage root number per plant, storage root weight, storage root weight per plant, and the expression of *IbAGPL3* in storage root (r > 0). Among them, the storage root number per plant (R^2^ = −0.69 *, *p* < 0.05) and storage root weight per plant (R^2^ = −0.90 **, *p* < 0.01) had a strong correlation with the expression of *IbAGPL3* in storage root.

## 3. Discussion

### 3.1. Evolution of IbSUSs and IbAGPases in Sweet potato

Through comparative genome analysis, the presence of *SUS* gene families has been detected in a variety of plant species. For instance, model species like *Arabidopsis thaliana* [21] and rice [22] are known to both contain six *SUS* genes. In our research, a comprehensive genome-wide analysis revealed nine *SUS* gene members in sweet potato, corroborating the findings of Jiang et al. [1]. Phylogenetic analysis of *SUS* genes from the sweet potato *Arabidopsis* and rice categorized these species into three groups (Figure 4A). The conserved motif analysis showed that IbSUS3 had seven motifs, while other IbSUSs contained eight motifs, with similar distribution patterns. Notably, IbSUS3 was homologous to IbSUS4 but lacked motif 6 (Figure 2B), indicating that IbSUS3 went through structure changes during evolution, which might lead to its functional differences. Predictions for secondary and tertiary structures suggested that SUS proteins mainly consisted of α-helix and random coil structures (Table 3), and high structural similarity was observed among SUS proteins in sweet potato (Figure 8). Furthermore, interchromosomal relationships (Figure 5) and evolutionary pressure information on IbSUS (Table 2) were analyzed. These aspects were not previously reported by Jiang et al. [1].

In recent decades, *AGPase* gene family members have been discovered in several plant species, including *Arabidopsis thaliana* [28], rice [29,30], and cassava [31]. Specifically, six *AGPases* (comprising four *APLs* and two *APSs*) have been found in *Arabidopsis thaliana* [28], seven (four *APLs* and three *APSs*) in rice [29,30], and nine in cassava (six *APLs* and three *APSs*) [31]. Through a comprehensive genome-wide analysis, nine *AGPase* genes were identified in sweet potato and categorized into two subfamilies. Phylogenetic analysis revealed that *AGPase* genes in sweet potato, *Arabidopsis*, and rice could be grouped into two categories based on homology [27]. Among these, IbAGPL5, IbAGPS2, and IbAGPS3 each contained seven conserved motifs, whereas the remaining six IbAGPases had eight conserved motifs distributed similarly. IbAGPL5 was homologous to IbAGPL2 but lacked motif 8. Similarly, IbAGPS2 and IbAGPS3 were homologous to IbAGPS1, with IbAGPS2 lacking motif 8 and IbAGPS3 missing motif 3 (Figure 2E). These findings suggest that IbAGPL5, IbAGPS2, and IbAGPS3 have undergone structural changes over the course of evolution, which may result in functional differences.

### 3.2. Response of IbSUSs and IbAGPases to Excessive Nitrogen Stress during Storage Root Development

Appropriate nitrogen fertilizer application could increase the number of grains per panicle and grain weight, enhancing final rice production [35]. However, excessive nitrogen application reduced 1000-grain weight and seed setting rate, leading to lower rice yield [36]. Excessive nitrogen also inhibited SUS activity and starch content in maize [37], and AGPase activity and starch content in buckwheat [32]. Previous research has shown that excessive nitrogen significantly suppresses the enzyme activity of SUS and AGPase, reducing starch content and grain weight in rice [9]. Similarly, in sweet potato, excessive nitrogen downregulated *AGPa* and *AGPb* expression, inhibited AGPase activity, and decreased starch content in roots [10,12]. It also reduced the number and weight of storage roots per plant [10] and fresh weight of developing storage roots [12], resulting in lower yield [13,16,38]. While appropriate nitrogen conditions upregulated SUS gene expression, increasing starch accumulation and total starch content in proso millet grains [25], excessive nitrogen decreased soluble sugar content in apple leaves by downregulating *MdSUS5* expression [26]. This study found that under excessive nitrogen stress, the expression of *IbAGPL1*, *IbAGPL2*, *IbAGPL4*, *IbAGPL5*, *IbAGPL6*, *IbAGPS1*, and *IbAGPS2* in fibrous root (Figure 9E), *IbSUS2*, *IbSUS6*, *IbSUS7*, *IbSUS8*, and *IbSUS9* in storage root (Figure 9F), and *IbAGPL2* and *IbAGPL4* in storage root (Figure 9G) was significantly (*p* < 0.05) downregulated. Moreover, the expression of *IbSUSs* and *IbAPGases* showed a different pattern. It is likely that EN may promote lateral roots, which are similar to excessive vegetative growth by EN, observed in most crop plants [39,40]. Developing lateral roots requires lots of energy, like sucrose, and thus, *IbSUSs* could be upregulated [41], which may assist the sweet potato in resisting the excessive nitrogen stress. Furthermore, excessive nitrogen significantly (*p* < 0.05) decreased SUS (sucrose decomposition direction, Figure 9B), AGPase (Figure 9C) activities, and starch content (Figure 9A), consistent with previous findings in rice [9]. Additionally, the number of storage roots under excessive nitrogen stress was lower than under normal nitrogen conditions (Figure 10). The results above indicated similar adverse effects, as previously reported [10,12,13,14].

In our investigation, we identified several homologous genes, including *IbAGPL3*, *OsAGPL3*, *OsAGPL1*, and *OsAGPL2* (Figure 4B). Earlier studies have reported that mutations in *OsAGPL3* and *OsAGPL1* significantly reduce starch production [42,43]. In mutants lacking *OsAGPL3*, both AGPase activity and starch levels in leaf blades were found to be less than 1% and 5% of those in wild-type blades, respectively [43]. The absence of *OsAGPL1* led to a 23% reduction in total AGPase activity within rice endosperm, along with minimal starch accumulation in culms and reduced starch content in mutant embryos [42]. Some observations underscore the essential role of *OsAGPL2* in the process of starch synthesis [30]. However, it was found that *IbAGPL*3 expression was the highest in storage roots when subjected to excessive nitrogen stress (Figure 9G), suggesting its involvement in the stress response mechanism. Moreover, there were strongly negative correlations among storage root number per plant, storage root weight per plant, and the expression of *IbAGPL3* in storage root. Given these insights, *IbAGPL3* emerges as a potential candidate for regulating starch synthesis and stress response to excessive nitrogen in sweet potato. Nonetheless, more research is required to fully understand its molecular mechanisms.

## 4. Materials and Methods

### 4.1. Identification of SUSs and AGPases

We acquired the entire set of protein sequences for sweet potato from the Ipomoea Genome Hub (https://ipomoea-genome.org/ (accessed on 2 April 2023)) and the Sweetpotato Genomics Resource (http://sweetpotato.uga.edu/ (accessed on 6 April 2023)). For the identification of all SUS and AGPase proteins in sweet potato, we utilized Hidden Markov Model (HMM) profiles to search for core domains, including sucrose synthase (PF00862), glycosyltransferase (PF00534), and ADP-glucose pyrophosphorylase (PF00483), obtained from the Pfam database (http://pfam.xfam.org/ (accessed on 6 April 2023)). Moreover, we incorporated sequences of six *SUS* and six *AGPase* genes from *Arabidopsis thaliana* through the Arabidopsis database (http://www.arabidopsis.org/ (accessed on 9 May 2023)).

### 4.2. Chromosomal Distribution and Gene Structure of SUSs and AGPases

Using chromosomal localization data from the Ipomoea Genome Hub (https://ipomoea-genome.org/ (accessed on 20 May 2023)) and the Sweetpotato Genomics Resource (http://sweetpotato.uga.edu/ (accessed on 20 May 2023)), we mapped *IbSUS* and *IbAGPase* genes individually onto sweet potato chromosomes. Visualization of gene structures was carried out using TBtools v2.096 [44].

### 4.3. Property Prediction of SUSs and AGPases

The attributes of SUS and AGPase proteins, including size, molecular weight (MW), isoelectric point (pI), instability index, and grand average of hydropathicity (GRAVY), were determined using the Expasy website (https://web.expasy.org/ (accessed on 26 May 2023)). Subcellular localization was predicted with WoLF PSORT (https://wolfpsort.hgc.jp/ (accessed on 29 May 2023)).

### 4.4. Domain Identification and Conserved Motif Analysis of SUSs and AGPases

We made use of of the MEME website (https://meme-suite.org/meme/ (accessed on 1 June 2023)) to analyze the conserved motifs of SUSs and AGPases and the maximum number of motif parameters was set to 8 [45]. The conserved domain structures of SUSs and AGPases were visualized using TBtools v2.096 [44].

### 4.5. Promoter Analyses of SUSs and AGPases

We examined cis-acting elements in the promoter regions of *SUS* and *AGPase* genes, approximately 2000 bp in length, using the PlantCARE database (http://bioinformatics.psb.ugent.be/webtools/plantcare/html/ (accessed on 5 June 2023)) [46]. The identified elements were then visualized using TBtools v2.096 [44].

### 4.6. Phylogenetic Analysis of SUSs and AGPases

We acquired gene sequences for *SUS* and *AGPase* from *Arabidopsis thaliana* and *Oryza sativa* through the National Center for Biotechnology Information (https://www.ncbi.nlm.nih.gov/ (accessed on 13 June 2023)). After aligning these sequences, we constructed two phylogenetic trees using MEGA11, applying the neighbor-joining method and 1000 bootstrap replicates [47]. The phylogenetic trees were further beautified using the Evolview online tool (http://evolgenius.info/#/ (accessed on 17 June 2023)).

### 4.7. Ka/Ks Analyses and Gene Collinearity of SUSs and AGPases

To calculate the Ka/Ks ratio [34], we assessed the nucleotide substitution parameters—Ks (synonymous) and Ka (nonsynonymous)—of the duplicated genes utilizing TBtools v2.096 [44]. Gene duplication events in sweet potato *SUS* and *AGPase* and collinearity analyses with *Ipomoea trifida* and *Ipomoea triloba* species were assessed and analyzed.

### 4.8. Protein Interaction Network Analysis of SUS and AGPase Regulated Proteins

We developed a protein interaction network for SUSs and AGPases using STRING (https://string-db.org (accessed on 11 July 2023)) based on homologous proteins from *Arabidopsis*, illustrating how these proteins interact.

### 4.9. Secondary and Tertiary Structure Analysis of SUS and AGPase Proteins

SOPMA (https://npsa-prabi.ibcp.fr/cgi-bin/npsa_automat.pl?page=npsa_sopma.html (accessed on 15 July 2023)) was employed to predict the secondary structures of SUS and AGPase proteins, whereas Phyre2 (http://www.sbg.bio.ic.ac.uk/phyre2/html/page.cgi?id=index (accessed on 18 July 2023)) was used for predicting their tertiary structures.

### 4.10. Pot Experiments and Plant Sampling

A randomized block design with three replications was used for the pot experiments. Experiments were carried out using the sweet potato variety “Pushu 32” in 2021 at the Agricultural Base of Hainan University, located at 20°3′39″ N latitude and 110°19′8″ E longitude. Fertilizers applied included P_2_O_5_, K_2_O, and micro-fertilizers. The control group received moderate N fertilization of 120 kg ha^−1^ (MN), while the treatment group received excessive N fertilization of 240 kg ha^−1^ (EN). Potassium and phosphate fertilizers were applied at levels of 240 kg K ha^−1^ and 120 kg P ha^−1^, respectively. Each treatment was subdivided into different plots.

Plant samples were collected at two time points: at 15 and 45 d after planting. For each plant, we mixed the fibrous roots at 15 d and storage roots at 45 d and cut them into 1 cm pieces, separately, then immediately froze them in liquid nitrogen and stored them at −80 °C for subsequent enzymatic activity assays and qRT-PCR analysis. At 15 d and 45 d, we selected five plants to sterilize at 105 °C and dried them at 60 °C. The dried samples were ground to a fine powder for starch content determination. Roots with diameters smaller than 0.2 cm were categorized as fibrous roots, while those exceeding 0.5 cm in diameter were classified as storage roots. The counts of fibrous roots at 15 d and storage roots at 45 d per plant were analyzed for differences between MN and EN treatments.

### 4.11. Starch Content in Fibrous Root and Storage Root

The starch content in both fibrous and storage roots, 15 and 45 days post-planting, was determined using the anthrone colorimetric method.

### 4.12. Sucrose Synthase (SUS) and ADP-Glucose Pyrophosphorylase (AGPase) Activity Assay

The sucrose synthase (SUS) and ADP-glucose pyrophosphorylase (AGPase) activity in both fibrous and storage roots were determined by visible spectrophotometry using sucrose synthase (decomposition direction, SS-I) and ADP-glucose pyrophosphorylase test kit produced by Beijing Solarbio Science&Technology Co., Ltd. (Beijing, China).

### 4.13. qRT-PCR Analysis of SUSs and AGPases

RNA was isolated from plant tissues using the operation method described in the instructions of the kit (RNAprep Pure Plant Plus Kit DP441), provided by Tiangen Biochemical Technology Co., Ltd. (Beijing, China). For cDNA synthesis, one microgram of the isolated RNA was reverse transcribed with HiScript II Q RT SuperMix (Nanjing, China). To determine the transcript levels of IbSUS and IbAGPase genes, qRT-PCR was carried out using gene-specific primers along with TB Green Premix Ex Taq II RR820A (Tokyo, Japan) on a qRT-PCR machine. The housekeeping gene β-actin served as an internal control, and the 2^−ΔΔCT^ method was utilized to compute relative expression levels. The experiment was performed with three independent biological replicates. The sequences of the primers used are listed in Table 5.

## 5. Conclusions

Excessive nitrogen inhibited starch synthesis and accumulation, which was not conducive to root development and storage root formation of sweet potato. The expression of a candidate gene, *IbAGPL3*, was strong negatively correlated with the number and weight of storage roots per plant, potentially responsive to excessive nitrogen stress, and associated with starch synthesis in sweet potato; this was identified through pot experiments, bioinformatics analysis, and qRT-PCR technology. This research provides fresh perspectives on the regulatory mechanisms of *IbSUS* and *IbAGPase* expression under excessive nitrogen stress and their role in the formation of storage roots in sweet potato.

## Figures and Tables

**Table 1:** Characteristics of *IbSUSs* and *IbAGPases*.

Gene ID	Gene Name	Protein Size (aa)	MW (Da)	*p*I	Instability Index	GRAVY	Subcellular Localization
*g505.t1*	*IbSUS1*	882	99,986.35	6.21	40.26	−0.312	cytosol
*g5497.t1*	*IbSUS2*	811	92,130.29	6.02	38.17	−0.275	chloroplast
*g29617.t1*	*IbSUS3*	837	94,763.23	6.14	36.59	−0.351	cytosol
*g30039.t1*	*IbSUS4*	839	94,952.6	6.33	38.73	−0.33	cytosol
*g31210.t1*	*IbSUS5*	743	84,781.48	6.11	36.16	−0.202	plasma membrane
*g55056.t1*	*IbSUS6*	763	87,821.85	6.17	33.53	−0.298	mitochondrion
*g55074.t1*	*IbSUS7*	851	97,494.1	6.16	38.91	−0.256	mitochondrion
*g55342.t1*	*IbSUS8*	906	101,580.88	6.99	39.82	−0.214	chloroplast
*g60893.t1*	*IbSUS9*	763	87,703.25	6.18	35.82	−0.261	cytosol
*g13324.t1*	*IbAGPL1*	827	91,001.22	9.08	35.39	−0.164	endoplasmic reticulum
*g34068.t1*	*IbAGPL2*	537	59,880.73	6.5	32.44	−0.173	cytosol
*g38633.t1*	*IbAGPL3*	496	55,273.67	8.37	30.71	−0.192	chloroplast
*g43092.t1*	*IbAGPL4*	484	53,623.32	5.93	34.01	−0.154	chloroplast
*g59752.t1*	*IbAGPL5*	501	55,690.41	6.37	37.47	−0.254	cytosol
*g60496.t1*	*IbAGPL6*	518	57,247.42	6.68	30.05	−0.184	cytoskeleton
*g30656.t1*	*IbAGPS1*	522	57,155.24	6.74	39.79	−0.178	cytosol
*g54359.t1*	*IbAGPS2*	520	56,904.82	7.58	37.85	−0.203	chloroplast_cytosol
*g55556.t1*	*IbAGPS3*	579	63,531.23	5.78	49.69	−0.287	chloroplast

Note: MW, molecular weight; *p*I, isoelectric point; GRAVY, grand average of hydropathicity.

**Table 2:** Evolution selection pressure information of *IbSUS* and *IbAGPase*.

Gene Pairs	Ka	Ks	Ka/Ks
*IbSUS1*/*IbSUS3*	0.102	0.5658	0.1803
*IbSUS6*/*IbSUS9*	0.0342	1.1358	0.0301
*IbSUS7*/*IbSUS5*	0.1389	1.0692	0.1299
*IbSUS3*/*IbSUS4*	0.017	0.0292	0.5796
*IbAGPS2*/*IbAGPS1*	0.049	0.4795	0.1021
*IbAGPL5*/*IbAGPL2*	0.1111	0.6767	0.1642

**Table 3:** The secondary structure of IbSUSs and IbAGPases.

Protein	α-Helix	β-Corner	Random Coil	Extended Strand
IbSUS1	51.13%	7.14%	29.59%	12.13%
IbSUS2	53.14%	6.54%	27.25%	13.07%
IbSUS3	49.22%	6.21%	32.50%	12.07%
IbSUS4	51.49%	6.56%	29.56%	12.40%
IbSUS5	48.99%	6.46%	30.96%	13.59%
IbSUS6	53.08%	7.08%	26.61%	13.24%
IbSUS7	53.35%	6.58%	26.67%	13.40%
IbSUS8	49.45%	6.84%	31.13%	12.58%
IbSUS9	52.95%	6.03%	28.44%	12.58%
IbAGPL1	35.91%	0.00%	49.09%	14.99%
IbAGPL2	20.67%	0.00%	59.40%	19.93%
IbAGPL3	20.97%	0.00%	57.46%	21.57%
IbAGPL4	20.87%	0.00%	58.26%	20.87%
IbAGPL5	21.16%	0.00%	57.88%	20.96%
IbAGPL6	19.96%	0.00%	61.20%	19.11%
IbAGPS1	20.88%	0.00%	58.81%	20.31%
IbAGPS2	20.38%	0.00%	60.77%	18.85%

**Table 4:** Agronomic characteristics of sweet potato at different days after planting.

Days after Planting	Nitrogen Level	FRNPP	FRWPP (g)	SRNPP	SRW (g)	SRWPP (g)
15 d	MN	28.50 ± 1.61 a	1.71 ± 0.23 a	-	-	-
	EN	24.00 ± 1.41 a	1.53 ± 0.24 a	-	-	-
45 d	MN	-	-	4.00 ± 0.45 a	20.11 ± 1.52 a	77.69 ± 4.60 a
	EN	-	-	2.50 ± 0.22 b	17.41 ± 2.41 a	41.27 ± 3.38 b

Note: One-way ANOVA, LSD. The values after the ± sign are the standard deviation; values followed by lowercase letters within a column are significantly different among treatments (*p* < 0.05). Data are expressed as the mean of six replications (*n* = 6). FRNPP, fibrous root number per plant; FRWPP, fibrous root weight per plant; SRNPP, storage root number per plant; SRW, storage root weight; SRWPP, storage root weight per plant.

**Table 5:** Primer sequences used in qRT-PCR.

Primer Name	Primers Sequence
*IbSUS1*	CCTTTTGTTCTTCCCATACCACTC
	CATACCTCTTTCACCTTGCCAG
*IbSUS2*	TCAGTGTACCGATGTGCTCATC
	TCTGGTTGTGGTTGGAGGTTC
*IbSUS3*	AGTGCGGTAGAGTTCGCTGTT
	TCTGGTTGTGGTTGGAGGTTC
*IbSUS4*	AGTGCGGTAGAGTTCGCTGTT
	TTATCATCACCAGCACTTTCCA
*IbSUS5*	GTCAGCCTCTTCTCCTTCTCAG
	ATGTTGTCATTCTTTCCCCCC
*IbSUS6*	CGCTTAATCATCTCACGCTCC
	GTTCACCAATACAAGGGCAAG
*IbSUS7*	GGGTTGGAGGGTCGTTAGATA
	TGTAGGAGTAGAGGAGGGGCG
*IbSUS8*	TAAGTTGGGCTGTGGTTTTGG
	ATAGCCTCCGTGAGCGTCTT
*IbSUS9*	ATCCCCGAGTTTTTCCTTGT
	TTGCCACCATCAGCCATCACTAAC
*IbAGPL1*	TTGCCACCATCAGCCATCACTAAC
	ATTCGAGACCGGATGCCAACAAC
*IbAGPL2*	TCTCCTCGATTCCTACCACCAACC
	CACCGAAATCTAACCGTGACCTCT
*IbAGPL3*	GTTCCCTCAATCGTCACCTTTCTCG
	GTGGCAGCAAGAACCTCAACAAATC
*IbAGPL4*	CAATGATGCTCTTGCTGTGGAACTG
	GCCGCCTCCCAGTATGATTGC
*IbAGPL5*	CCGAGATTCTTCCTGCTGCTGTG
	ATTGTCCCGATGTCCTCCCAGTAG
*IbAGPL6*	GGTGACGCCAAGAACAAGAACATTG
	GCTCGGCTATCATCCATTGGTACAC
*IbAGPS1*	ATGTTAGCAAGGCGTCGTTCCG
	CGAGGAGGCAATGGCATCAGC
*IbAGPS2*	GCATCACTCAATCGTCACCTCTCAC
	GCCTCACAGCATCAGCAGTACC
*IbAGPS3*	CCGCTATACACTTTACCTCGCCATC
	CCCTTCTCCCACCCTTGTCCTC

## Data Availability

Data are available on request due to privacy or ethical restrictions.

## References

[1] Jiang Z., Zhang H., Gao S., Zhai H., He S., Zhao N., Liu Q. (2023). Genome-wide identification and expression analysis of the sucrose synthase gene family in sweet potato and its Two Diploid Relatives. Int. J. Mol. Sci..

[2] Nie H., Kim S., Kim J., Kwon S.Y., Kim S.H. (2022). Comparative analysis of AGPase proteins and conserved domains in sweetpotato (*Ipomoea batatas* (L.) Lam.) and its two wild relatives. J. Plant Biotechnol..

[3] Firon N., LaBonte D., Villordon A., Kfir Y., Solis J., Lapis E., Perlman T.S., Doron-Faigenboim A., Hetzroni A., Althan L. (2013). Transcriptional profiling of sweetpotato (*Ipomoea batatas*) roots indicates down-regulation of lignin biosynthesis and up-regulation of starch biosynthesis at an early stage of storage root formation. BMC Genom..

[4] Yang Y., Zhu J., Sun L., Kong Y., Chen J., Zhu M., Xu T., Li Z., Dong T. (2023). Progress on physiological and molecular mechanisms of storage root formation and development in sweetpotato. Sci. Hortic..

[5] Lyu R., Ahmed S., Fan W., Yang J., Wu X., Zhou W., Zhang P., Yuan L., Wang H. (2021). Engineering properties of sweet potato starch for industrial applications by biotechnological techniques including genome editing. Int. J. Mol. Sci..

[6] Ravi V., Indira P. (1998). Crop physiology of sweetpotato. Hortic. Rev.

[7] Glibert P.M., Harrison J., Heil C., Seitzinger S. (2006). Escalating worldwide use of urea–a global change contributing to coastal eutrophication. Biogeochemistry.

[8] Zhang Y., Liu R., Liu Z., Hu Y., Xia Z., Hu B., Rennenberg H. (2024). Consequences of excess urea application on photosynthetic characteristics and nitrogen metabolism of *Robinia pseudoacacia* seedlings. Chemosphere.

[9] Chen Y., Teng Z., Yuan Y., Yi Z., Zheng Q., Yu H., Lv J., Wang Y., Duan M., Zhang J. (2022). Excessive nitrogen in field-grown rice suppresses grain filling of inferior spikelets by reducing the accumulation of cytokinin and auxin. Field Crop. Res..

[10] Duan W., Zhang H., Xie B., Wang B., Zhang L. (2019). Impacts of nitrogen fertilization rate on the root yield, starch yield and starch physicochemical properties of the sweet potato cultivar Jishu 25. PLoS ONE.

[11] Chen X., Ding Y., Tang Z., Wei M., Shi X., Zhang A., Li H. (2015). Suitable nitrogen rate for storage root yield and quality of sweet potato. J. Plant Nutr. Fertil..

[12] Duan W., Zhang H., Wang Q., Xie B., Zhang L. (2023). Regulation of root development in nitrogen-susceptible and nitrogen-tolerant sweet potato cultivars under different nitrogen and soil moisture conditions. BMC Plant Biol..

[13] Taranet P., Harper S., Kirchhof G., Fujinuma R., Menzies N. (2017). Growth and yield response of glasshouse-and field-grown sweetpotato to nitrogen supply. Nutr. Cycl. Agroecosystems.

[14] Fernandes A.M., Ribeiro N.P., Assunção N.S., Nunes J.G.d.S., Sorroche C.P., Leonel M. (2021). Impact of nitrogen and green manure on yield and quality of sweet potato in sandy soil: A Brazilian case study. J. Agric. Food Res..

[15] Duan W., Zhang H., Xie B., Wang B., Hou F., Li A., Dong S., Qin Z., Wang Q., Zhang L. (2021). Nitrogen utilization characteristics and early storage root development in nitrogen-tolerant and nitrogen-susceptible sweet potato. Physiol. Plant..

[16] Du X., Xi M., Kong L. (2019). Split application of reduced nitrogen rate improves nitrogen uptake and use efficiency in sweetpotato. Sci. Rep..

[17] Keller M. (2014). Investigation of Cassava Storage Root Initiation and Development for Engineering Increases in Starch and Storage Root Yield. Ph.D. Thesis.

[18] Petreikov M., Eisenstein M., Yeselson Y., Preiss J., Schaffer A.A. (2010). Characterization of the AGPase large subunit isoforms from tomato indicates that the recombinant L3 subunit is active as a monomer. Biochem. J..

[19] Li X.Q., Zhang D. (2003). Gene expression activity and pathway selection for sucrose metabolism in developing storage root of sweet potato. Plant Cell Physiol..

[20] Chen A., He S., Li F., Li Z., Ding M., Liu Q., Rong J. (2012). Analyses of the sucrose synthase gene family in cotton: Structure, phylogeny and expression patterns. BMC Plant Biol..

[21] Baud S., Vaultier M.N., Rochat C. (2004). Structure and expression profile of the sucrose synthase multigene family in *Arabidopsis*. J. Exp. Bot..

[22] Hirose T., Scofield G.N., Terao T. (2008). An expression analysis profile for the entire sucrose synthase gene family in rice. Plant Sci..

[23] Horst I., Welham T., Kelly S., Kaneko T., Sato S., Tabata S., Parniske M., Wang T.L. (2007). TILLING mutants of *Lotus japonicus* reveal that nitrogen assimilation and fixation can occur in the absence of nodule-enhanced sucrose synthase. Plant Physiol..

[24] Angeles-Núñez J.G., Tiessen A. (2010). *Arabidopsis* sucrose synthase 2 and 3 modulate metabolic homeostasis and direct carbon towards starch synthesis in developing seeds. Planta.

[25] Wang H., Zhang H., Liu J., Ma Q., Wu E., Gao J., Yang Q., Feng B. (2024). Transcriptome analysis reveals the mechanism of nitrogen fertilizers in starch synthesis and quality in waxy and non-waxy proso millet. Carbohydr. Polym..

[26] Wang F., Ge S., Xu X., Xing Y., Du X., Zhang X., Lv M., Liu J., Zhu Z., Jiang Y. (2021). Multiomics analysis reveals new insights into the apple fruit quality decline under high nitrogen conditions. J. Agric. Food Chem..

[27] Batra R., Saripalli G., Mohan A., Gupta S., Gill K.S., Varadwaj P.K., Balyan H.S., Gupta P.K. (2017). Comparative analysis of AGPase genes and encoded proteins in eight monocots and three dicots with emphasis on wheat. Front. Plant Sci..

[28] Crevillén P., Ventriglia T., Pinto F., Orea A., Mérida Á., Romero J.M. (2005). Differential pattern of expression and sugar regulation of *Arabidopsis thaliana* ADP-glucose pyrophosphorylase-encoding genes. J. Biol. Chem..

[29] Lu F.H., Park Y.J. (2012). Sequence variations in OsAGPase significantly associated with amylose content and viscosity properties in rice (*Oryza sativa* L.). Genet. Res..

[30] Tang X., Peng C., Zhang J., Cai Y., You X., Kong F., Yan H., Wang G., Wang L., Jin J. (2016). ADP-glucose pyrophosphorylase large subunit 2 is essential for storage substance accumulation and subunit interactions in rice endosperm. Plant Sci..

[31] Wang M., Xiao X., Ying D., Wang Q., Li L., Zhang R., Ye J. (2019). Bioinformatics and expression of AGPase gene family in cassava. J. South. Agric..

[32] Gao L., Wang H., Wan C., Wang P., Eeckhout M., Gao J. (2023). Suitable nitrogen fertilizer application drives the endosperm development and starch synthesis to improve the physicochemical properties of common buckwheat grain. Int. J. Biol. Macromol..

[33] Zhou Y., Chen Y., Tao X., Cheng X., Wang H. (2016). Isolation and characterization of cDNAs and genomic DNAs encoding ADP-glucose pyrophosphorylase large and small subunits from sweet potato. Mol. Genet. Genom..

[34] Meng Y., Wang N., Zhang H., Xu R., Si C. (2023). Genome-wide analysis of sweet potato ammonium transporter (AMT): Influence on nitrogen utilization, storage root development and yield. Int. J. Mol. Sci..

[35] Jiang Q., Du Y., Tian X., Wang Q., Xiong R., Xu G., Yan C., Ding Y. (2016). Effect of panicle nitrogen on grain filling characteristics of high-yielding rice cultivars. Eur. J. Agron..

[36] Zhao C., Liu G., Chen Y., Jiang Y., Shi Y., Zhao L., Liao P., Wang W., Xu K., Dai Q. (2022). Excessive nitrogen application leads to lower rice yield and grain quality by inhibiting the grain filling of inferior grains. Agriculture.

[37] Singletary G.W., Doehlert D.C., Wilson C.M., Muhitch M.J., Below F.E. (1990). Response of enzymes and storage proteins of maize endosperm to nitrogen supply. Plant Physiol..

[38] Bellini C., Pacurar D.I., Perrone I. (2014). Adventitious roots and lateral roots: Similarities and differences. Annu. Rev. Plant Biol..

[39] Shi Z., Fan X., Klaus D., Sattemacher B. (2005). Effect of localized nitrogen supply on root morphology in rice and its mechanism. Chin. J. Rice Sci..

[40] Zhang L., Sun S., Liang Y., Li B., Ma S., Wang Z., Ma B., Li M. (2021). Nitrogen levels regulate sugar metabolism and transport in the shoot tips of crabapple plants. Front. Plant Sci..

[41] Duan W., Wang Q., Zhang H., Xie B., Li A., Hou F., Dong S., Wang B., Qin Z., Zhang L. (2018). Comparative study on carbon–nitrogen metabolism and endogenous hormone contents in normal and overgrown sweetpotato. S. Afr. J. Bot..

[42] Cook F.R., Fahy B., Trafford K. (2012). A rice mutant lacking a large subunit of ADP-glucose pyrophosphorylase has drastically reduced starch content in the culm but normal plant morphology and yield. Funct. Plant Biol..

[43] Rösti S., Fahy B., Denyer K. (2007). A mutant of rice lacking the leaf large subunit of ADP-glucose pyrophosphorylase has drastically reduced leaf starch content but grows normally. Funct. Plant Biol..

[44] Chen C., Wu Y., Li J., Wang X., Zeng Z., Xu J., Liu Y., Feng J., Chen H., He Y. (2023). TBtools-II: A “one for all, all for one” bioinformatics platform for biological big-data mining. Mol. Plant.

[45] Bailey T.L., Johnson J., Grant C.E., Noble W.S. (2015). The MEME suite. Nucleic Acids Res..

[46] Lescot M., Déhais P., Thijs G., Marchal K., Moreau Y., Peer Y.V.d., Rouzé P., Rombauts S. (2002). PlantCARE, a database of plant cis-acting regulatory elements and a portal to tools for in silico analysis of promoter sequences. Nucleic Acids Res..

[47] Thompson J.D., Gibson T.J., Plewniak F., Jeanmougin F., Higgins D.G. (1997). The CLUSTAL_X windows interface: Flexible strategies for multiple sequence alignment aided by quality analysis tools. Nucleic Acids Res..

